# Characteristics of a Pure Acute Subdural Hematoma Caused by Intracranial Aneurysm Rupture: A Case Report and Review of Literature

**DOI:** 10.7759/cureus.66575

**Published:** 2024-08-10

**Authors:** Soshi Gotan, Susumu Yamaguchi, Michiharu Yoshida, Mitsuto Iwanaga, Tsuyoshi Izumo, Takayuki Matsuo

**Affiliations:** 1 Department of Neurosurgery, Sasebo City General Hospital, Sasebo, JPN; 2 Department of Neurosurgery, Sasebo City General Hospital, Sasebo, Japan; 3 Department of Neurosurgery, Nagasaki University Graduate School of Medicine, Nagasaki, JPN

**Keywords:** outcomes, rebleeding, delayed diagnosis, aneurysm rupture, acute subdural hematoma

## Abstract

Pure acute subdural hematomas (ASDHs) due to ruptured aneurysms without subarachnoid or intracerebral hemorrhage are rare. We report the case of a 26-year-old female who presented with a pure ASDH caused by a ruptured distal anterior cerebral artery (ACA). The patient complained of sudden headache and vomiting and was transferred to our hospital. On the ambulance journey to the hospital, her consciousness level decreased suddenly just after experiencing additional pain in the head. At admission, the consciousness level was 4 points on the Glasgow coma scale with bilateral pupil dilatation. Computed tomography (CT) and CT angiography showed a left ASDH without subarachnoid hemorrhage (SAH) and a distal ACA aneurysm. Emergent hematoma evacuation was performed, but SAH and the bleeding point were not observed. Therefore, coil embolization for the distal ACA aneurysm was performed after an emergent operation. During embolization, intraoperative rupture was observed. The contrast media was seen up to the convexity subdural space along the falx. Extravasation ceased after intraaneurysmal coil embolization. Consequently, the rupture of the distal ACA aneurysm was diagnosed as the cause of the pure ASDH. The patient received additional coil embolization due to recanalization of the aneurysm without rebleeding 44 days after admission and was transferred to a rehabilitation hospital 55 days after admission to our hospital with a score of 4 on the modified ranking scale. From the reviews of 56 patients from 32 studies, including our case, we determine that an ACA aneurysm could show the distant hematomas located far from the site of a ruptured aneurysm compared with a ruptured aneurysm located in the internal carotid and middle cerebral arteries. Distant hematoma location could also lead to delayed diagnosis of aneurysms and lead to rebleeding and poor outcomes. Aneurysm rupture diagnoses should receive special attention, especially for ACA aneurysms, as the hematoma may be located far from the rupture site.

## Introduction

Acute subdural hematomas (ASDHs) are commonly caused by head trauma, whereas nontraumatic or spontaneous SDHs are rare, accounting for 0.7-6.7% of all ASDHs [1]. Although aneurysm rupture usually causes subarachnoid hemorrhage (SAH), ASDHs have been reported in 1.2% of patients with nontraumatic SAH [2] and in 0.5-7.9% of all cases of aneurysm rupture [3,4]. However, ASDHs caused by aneurysm rupture without SAH, known as pure ASDHs, are rare and were reported in 2.9% of cases of ruptured aneurysms in an autopsy series [5]. Due to their rarity, the characteristics of pure ASDHs caused by an aneurysm rupture are not well documented.

Here, we report a case of pure ASDH caused by a ruptured distal anterior cerebral artery (ACA) aneurysm and review the literature concerning pure ASDH to reveal the characteristics of pure ASDHs caused by an aneurysm rupture.

## Case presentation

**Figure 1:**
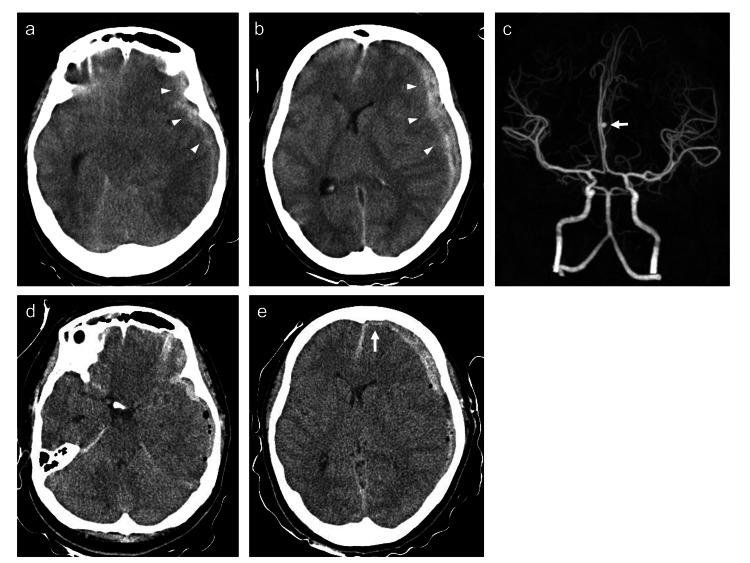
Imaging findings based on our patient with a pure acute subdural hematoma (ASDH) before the operation. (a, b) Axial computed tomography (CT) images obtained on admission showing a left convexity ASDH (arrowheads) with uncal herniation, with no evidence of subarachnoid hemorrhage (SAH). (c) CT angiography image showing a 4 mm right distal anterior cerebral artery (ACA) aneurysm (arrow). (d, e) CT just after hematoma evacuation using burr hole showing improvement of uncal herniation without SAH and connectivity of ASDH between interhemispheric fissure and left convexity space (arrow).

**Figure 2:**
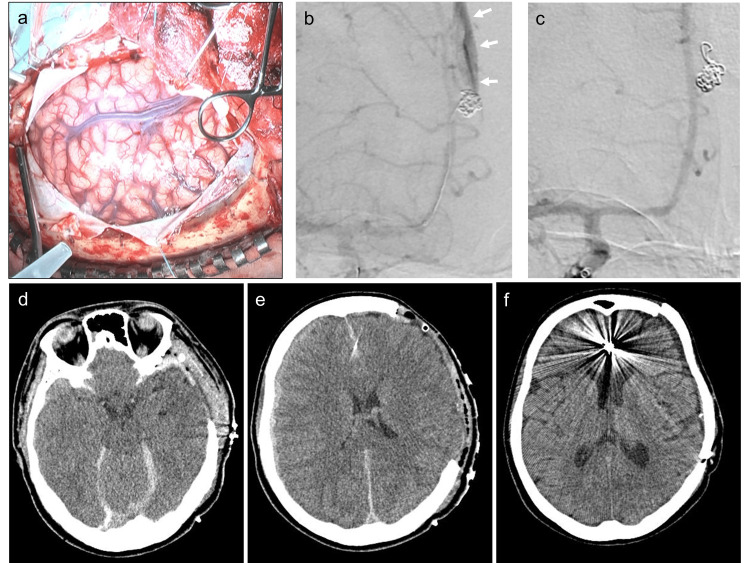
Intraoperative and postoperative imaging findings. (a) Intraoperative image showing no evidence of subarachnoid hemorrhage (SAH) or vessel injury that could cause the acute subdural hematoma (ASDH). (b) Internal carotid arteriography image showing extravasation (arrows), with the upward migration of the contrast medium along the falx. (c) Follow-up internal carotid arteriography image obtained after the completion of coil embolization showing complete aneurysm occlusion. (d, e) Computed tomography (CT) images just after coil embolization showing increased ASDH containing contrast medium around cerebellar tentorium, right convexity, and interhemispheric fissure adjacent to coil mass with no evidence of SAH. (f) CT one day after additional coil embolization showing no new lesion.

A 26-year-old woman presented with consciousness disturbances following a sudden, severe headache and vomiting. On admission, her Glasgow coma scale score was 4 (E1V1M2), and bilateral pupil dilation was observed. CT showed a left convexity ASDH with uncal herniation, with no evidence of an SAH (Figure 1a, 1b). CT angiography showed a 4-mm right distal ACA aneurysm (Figure 1c). Following this, emergent hematoma evacuation using one burr hole was performed in the emergency room. CT revealed improvement of uncal herniation without any evidence of SAH (Figure 1d), and connectivity of ASDH between interhemispheric space and left convexity (Figure 1e). Intraoperatively, we also discovered no evidence of an SAH and no vessel injury that could cause the ASDH (Figure 2a). Therefore, we performed coil embolization of the right distal ACA aneurysm following hematoma evacuation with a diagnosis of World Federation Neurosurgical Societies grade 5. During the second coil insertion, coil migration into the extra-aneurysmal space was observed. Internal carotid arteriography showed extravasation, with upward migration of the contrast medium along the falx (Figure 2b). Coil insertion was then continued, and the extravasation resolved. After the placement of three coils (total length, 18 cm), a complete aneurysm occlusion was observed (Figure 2c). Although CT performed just after coil embolization showed increased ASDH containing contrast medium around cerebellar tentorium (Figure 2d), right convexity, and interhemispheric space (Figure 2e), there was no SAH (Figure 2d, 2e). Thirty-seven days after admission, recanalization of the aneurysm was confirmed. Despite the absence of evidence of rebleeding, 44 days after admission, coil embolization of the aneurysm was performed, and CT one day after additional coil embolization showed no new lesion (Figure 2f). On day 55 after admission, the patient was transferred to a rehabilitation hospital with a modified Rankin Scale score of 4.

## Discussion

We encountered a case of pure ASDH caused by the rupture of a distal ACA aneurysm. Given that the ASDH was located far from the rupture site, determining its cause was challenging. Such cases may lead to a delayed diagnosis of aneurysm rupture and, consequently, poor outcomes. In cases of aneurysmal SAHs, an early diagnosis and treatment are crucial for preventing rebleeding [6]. Therefore, this study reviewed existing cases of pure ASDHs caused by the rupture of intracranial aneurysms to discern their characteristics and identify factors related to mortality.

We searched PubMed and Google Scholar for studies published in English and Japanese using the search terms “pure ASDH” and “ASDH and aneurysm.” All studies that mentioned the aforementioned terms were retrieved. In addition, the references of the retrieved studies were reviewed. Studies that did not include patient imaging data (computed tomography or magnetic resonance imaging) and outcomes, as well as cases that did not meet the criteria for pure ASDH were excluded. The following demographic, clinical, and imaging data were collected: age, sex, episode indicating aneurysm rupture within one month from admission, rebleeding in prehospital setting, delayed diagnosis of aneurysm rupture, aneurysm location, ASDH location, time from onset to admission, time from onset to aneurysm diagnosis, time from admission to aneurysm treatment, treatment method for aneurysmal obliteration, rebleeding rate after admission, and mortality rate. Sudden headaches, seizures, or changes in neurological status, including the level of consciousness, were considered episodes indicating aneurysm rupture. Cases where aneurysms were not diagnosed on the day of the visit (or within 24 h) were defined as delayed diagnosis. Distant hematoma was defined when the main or thickest hematoma was not adjacent to and far from the ruptured aneurysm. Regarding patient outcomes, we could only assess whether the patient was alive or dead. Two neurosurgeons (S.G. and S.Y.) independently assessed all data, including imaging findings. If there was a disagreement, a third neurosurgeon (M.Y.) made the final decision.

In the results, overall, 32 studies were found in the PubMed and Google Scholar databases. In addition, among the references, 40 studies were considered to be related to pure ASDHs or ASDHs with aneurysms. Among 72 studies, 54 patients from 45 studies met our inclusion criteria. The characteristics of the patients, including our patient, are presented in Table 1 [2,3,7-49].

**Table 1:** Characteristics of the included cases GCS: Glasgow coma scale; PHT: prehospital head trauma; ERA: episode indicating aneurysm rupture; PR: prehospital rebleed; DD: delayed diagnosis; WFNS: World Federation Neurosurgical Societies; OTA: onset-to-admission time; OTD; onset-to-diagnosis time; OTT: onset-to-treatment time; ATT: admission-to-treatment time; ICA: internal carotid artery; MCA: middle cerebral artery; dACA: distal anterior cerebral artery; Pcom: posterior communicating artery; IC-PC: internal carotid artery-posterior communicating artery; Acom: anterior communicating artery; D: direct surgery; E: endovascular treatment; O: observation; N/A: not available.

No.	First author	Age	Sex	GCS	PHT	ERA	PR	DD	WFNS on admission	Location	Hematoma	OTA (day)	OTD (day)	OTT (day)	ATT (day)	Treatment	Rebleed	Death
1	Rengachary, 1981 [7]	49	M	11	Yes	No	No	Yes	4	MCA	Adjacent	14	14	N/A	N/A	D	No	No
2	Williams, 1983 [8]	18	F	N/A	N/A	Yes	No	No	N/A	ICA	Adjacent	0	0	7	7	D	No	No
3	O'Leary, 1986 [9]	28	F	N/A	No	Yes	Yes	No	N/A	MCA	Adjacent	0	2	4	2	O	Yes	Yes
4	Kondziolka, 1988 [10]	43	M	4	No	Yes	N/A	No	5	Pcom	Adjacent	0	0	10	10	D	No	No
5		38	F	N/A	No	Yes	Yes	No	4	Pcom	Adjacent	14	14	16	2	D	No	No
6	Watanabe, 1991 [11]	51	M	4	No	Yes	No	Yes	5	dACA	Adjacent	0	2	4	2	O	Yes	Yes
7	Hashizume, 1992 [12]	67	M	14	N/A	Yes	No	No	3	dACA	Adjacent	0	0	0	0	D	Yes	No
8	Ragland, 1993 [13]	27	M	N/A	No	Yes	Yes	Yes	5	Acom	Far	0	1	8	7	O	Yes	Yes
9	Hatayama, 1994 [14]	55	M	15	N/A	Yes	Yes	No	1	dACA	Adjacent	0	0	0	0	D	No	No
10		66	F	N/A	Yes	Yes	No	No	5	dACA	Adjacent	0	0	0	0	D	Yes	No
11	Ishibashi, 1997 [15]	54	F	15	No	Yes	No	No	1	IC-PC	Adjacent	0	0	1	1	D	No	No
12	Huang, 1999 [16]	61	F	15	N/A	Yes	N/A	No	1	MCA	Adjacent	0	1	N/A	N/A	D	No	No
13	Satoh, 1999 [17]	58	F	N/A	N/A	Yes	No	Yes	4	ICA	Adjacent	0	0	51	51	D	Yes	No
14		25	F	15	N/A	Yes	N/A	No	1	IC-PC	Adjacent	0	0	1	1	D	No	No
15		22	F	3	N/A	Yes	N/A	No	5	IC-PC	Adjacent	0	0	0	0	D	No	No
16	Ishikawa, 2000 [18]	62	M	15	N/A	Yes	No	No	1	IC-PC	Adjacent	10	10	10	0	D	No	No
17	Nonaka, 2000 [19]	52	F	4	No	Yes	Yes	No	5	IC-PC	Adjacent	5	5	5	0	D	No	No
18	Araki, 2002 [20]	55	F	4	N/A	Yes	Yes	No	5	IC-PC	Adjacent	10	10	10	0	D	No	No
19	Inamasu, 2002 [2]	28	F	5	No	Yes	No	Yes	5	IC-PC	Adjacent	0	5	5	5	O	Yes	Yes
20	Nozar, 2002 [3]	56	M	7	No	Yes	No	Yes	4	Acom	Far	0	2	4	2	O	Yes	Yes
21		28	M	15	No	Yes	No	No	1	Pcom	Adjacent	0	0	1	1	D	No	No
22		39	F	N/A	N/A	Yes	No	No	N/A	Pcom	Adjacent	0	0	0	0	D	No	No
23		46	M	4	No	Yes	N/A	No	5	MCA	Adjacent	N/A	N/A	N/A	N/A	D	No	Yes
24	Katsuno, 2003 [21]	63	F	15	No	Yes	N/A	No	1	dACA	Adjacent	0	0	0	0	D	No	No
25	Ninomiya, 2003 [22]	51	M	4	No	Yes	Yes	No	5	dACA	Adjacent	0	0	6	6	D	No	No
26	Krishnaney, 2004 [23]	42	F	14	N/A	Yes	Yes	No	2	Acom	Adjacent	0	0	6	6	D	No	No
27	Hori, 2005 [24]	57	M	N/A	No	Yes	No	No	N/A	MCA	Adjacent	0	0	0	0	D	No	No
28	Kato, 2005 [25]	54	F	4	N/A	Yes	No	Yes	4	dACA	Far	0	N/A	N/A	N/A	D	No	No
29		65	F	6	N/A	N/A	N/A	Yes	5	dACA	Far	1	N/A	N/A	N/A	D	No	No
30	Koerbel, 2005 [26]	62	F	N/A	No	Yes	N/A	No	N/A	ICA	Far	0	1	2	3	E	Yes	No
31	Marinelli, 2005 [27]	65	F	15	No	Yes	Yes	No	1	IC-PC	Adjacent	9	9	9	0	E	No	No
32	Boujemâa, 2006 [28]	44	F	N/A	No	Yes	No	No	N/A	IC-PC	Far	0	0	0	0	E	No	Yes
33	Gilad, 2007 [29]	47	M	15	No	Yes	N/A	No	1	Acom	Adjacent	7	7	7	0	E	No	No
34	Brock, 2010 [30]	42	F	15	N/A	No	Yes	No	1	ICA	Adjacent	11	11	N/A	N/A	D	No	No
35	De Blasi, 2010 [31]	47	F	15	No	Yes	No	Yes	1	IC-PC	Adjacent	1	4	5	4	E	No	No
36		60	F	15	N/A	Yes	N/A	No	1	MCA	Adjacent	5	6	6	0	D	No	No
37	Kurabe, 2010 [32]	75	M	15	N/A	N/A	No	Yes	1	MCA	Adjacent	10	10	10	0	D	No	N/A
38	Weil, 2010 [33]	51	F	4	No	Yes	N/A	No	5	dACA	Adjacent	0	0	0	0	D	No	No
39	Takada, 2012 [34]	54	M	15	No	Yes	Yes	No	1	dACA	Adjacent	14	14	15	1	D	No	No
40	Mrfka, 2013 [35]	47	F	15	No	Yes	Yes	Yes	1	Pcom	Adjacent	7	10	10	3	E	Yes	No
41	Sumioka, 2013 [36]	69	F	15	No	Yes	No	No	1	dACA	Adjacent	0	0	1	1	D	No	No
42	Gong, 2014 [37]	43	M	15	No	Yes	No	Yes	1	MCA	Adjacent	8	16	16	8	D	Yes	No
43	Mansour, 2014 [38]	51	M	7	No	Yes	No	Yes	4	Pcom	Adjacent	0	1	1	1	E	No	No
44	Shepherd, 2014 [39]	48	M	N/A	No	Yes	No	No	5	ICA	Adjacent	0	0	0	0	E	Yes	No
45	Singla, 2014 [40]	25	F	10	No	No	No	No	4	MCA	Adjacent	0	0	0	0	D	No	No
46	Suyama, 2014 [41]	92	F	14	No	Yes	No	No	3	IC-PC	Adjacent	0	0	0	0	E	No	No
47	Awaji, 2016 [42]	43	M	15	No	Yes	No	No	1	MCA	Adjacent	0	0	0	0	D	Yes	No
48	Han, 2016 [43]	42	F	15	No	Yes	Yes	No	1	MCA	Adjacent	0	0	1	1	D	No	No
49	Lee, 2016 [44]	37	F	4	No	Yes	No	No	5	ICA	Adjacent	0	0	0	0	D	No	No
50	Song, 2016 [45]	48	F	15	No	Yes	No	No	1	dACA	Far	0	0	0	0	D	No	No
51	Sasaki, 2018 [46]	71	F	14	No	Yes	No	No	2	IC-PC	Adjacent	0	0	0	0	D	No	No
52	Hayashi, 2019 [47]	41	F	N/A	No	No	No	No	N/A	IC-PC	Adjacent	0	0	0	0	E	No	No
53	Al-Abdulwahhab, 2020 [48]	34	F	15	No	Yes	Yes	No	1	Pcom	Adjacent	2	2	2	0	D	No	No
54	Oka, 2020 [49]	73	F	15	No	Yes	No	No	1	IC-PC	Adjacent	0	0	0	0	D	Yes	No
55	Present case	26	F	4	No	Yes	Yes	No	5	dACA	Far	0	0	1	1	E	No	No

The mean patient age was 49.0 years, and 19 (34.5%) were men. The most common aneurysm location was the internal carotid artery (ICA; n = 20), followed by the ACA (n = 17), middle cerebral artery (n = 11), and posterior communicating artery (n = 7). No patients had posterior circulation aneurysms. Only two of the 40 (5.0%) patients had episodes of prehospital head trauma, and 49 of the 53 (92.5%) patients had episodes indicating aneurysm rupture. Delayed diagnosis of aneurysm rupture was noted in 11 patients. Regarding treatment, 38 and 11 patients underwent clipping and coil embolization, respectively. The in-hospital rebleeding and mortality rates were 25.5% (n = 14) and 13.0% (7/54), respectively. Among those who died, five (71.4%) patients experienced rebleeding during hospitalization.

Factors related to in-hospital mortality

This analysis included 54 patients because outcome data for one patient were unavailable (Table 2). The death group had lower Glasgow coma scale scores on admission than those of the survival group, thus indicating a poorer level of consciousness (median, 4.5 vs. 15). Furthermore, in the death group, we observed a higher proportion of patients with delayed aneurysm diagnosis (57.1% vs. 17.0%), a lower proportion of patients who underwent direct and endovascular treatment (28.6% vs. 100%), and a higher in-hospital rebleeding rate (71.4% vs. 19.1%) when compared to those in the survival group.

**Table 2:** Characteristics of patients with pure ASDHs caused by aneurysm rupture according to the survival outcome GCS: Glasgow coma scale; OTA: onset-to-admission time; OTD; onset-to-diagnosis time; OTT: onset-to-treatment time; ATT: admission-to-treatment time; ICA: internal carotid artery; MCA: middle cerebral artery; ACA: anterior cerebral artery; Pcom: posterior communicating artery.

Variable	Death (n = 7)	Alive (n = 47)
Age median (25-75%)	44 (28.0-51.0)	51 (41.0-61.0)
Sex (male)	4	14
GCS at admission	4.5 (4.0-6.5)	15 (5.0-15.0)
Prehospital head trauma		
Present	0	2
Absent	7	30
Unknown (missing)	0	15
Episode indicating the rupture of aneurysm		
Present	7	42
Absent	0	4
Unknown (missing)	0	1
Prehospital rebleed		
Present	2	13
Absent	4	24
Unknown (missing)	1	10
Delayed diagnosis		
Present	4	8
Absent	3	39
Location		
ICA	2	18
MCA	2	8
ACA	3	14
Pcom	0	7
Hematoma location		
Adjacent to the ruptured aneurysm	4	42
Far from the ruptured aneurysm	3	5
OTA (day) median	0.0 (0.0-0.0)	0.0 (0.0-5.0)
OTD (day) median (25%-75%)	2.0 (0.6-2.8)	0.0 (0.0-5.5)
OTT (day) median (25%-75%)	4.0 (3.0-5.8)	1.0 (0.0-7.0)
ATT (days) median (25%-75%)	2.0 (1.5-5.5)	0.0 (0.0-1.3)
Treatment		
Direct surgery	1	37
Endovascular treatment	1	10
Observation	5	0
Rebleeding after admission		
Present	5	9
Absent	2	38

Aneurysm location characteristics

This analysis included 56 patients. Their clinical characteristics according to the aneurysm location are presented in Tables 3-6. The ICA group had a lower proportion of men (10.0% vs. 48.5%, Table 3), the posterior communicating artery group had a lower median age (39.0 vs. 51.0 years, Table 6), and the ACA group had a lower proportion of cases where the aneurysm and ASDH were adjacent (64.7% vs. 94.7%, Table 5) than those of the other aneurysm location groups.

**Table 3:** ICA group GCS: Glasgow coma scale; OTA: onset-to-admission time; OTD; onset-to-diagnosis time; OTT: onset-to-treatment time; ATT: admission-to-treatment time; ICA: internal carotid artery; N/A not available.

Variable	ICA	
	Present (20)	Absent (35)
Age median (25%-75%)	50.0 (38.0-62.0)	49.0 (42.0-57.0)
Sex (male)	2	17
GCS at admission	15.0 (4.0-15.0)	14.0 (4.0-15.0)
Prehospital head trauma		
Present	0	2
Absent	13	25
Unknown (missing)	7	8
Episode indicating the rupture of aneurysm		
Present	18	32
Absent	2	2
Unknown (missing)	0	1
Prehospital rebleed		
Present	4	11
Absent	13	16
Unknown (missing)	3	0
Delayed diagnosis		
Present	3	9
Absent	17	26
Unknown (missing)	0	0
Hematoma location		
Adjacent to the ruptured aneurysm	18	29
Far from the ruptured aneurysm	2	6
OTA (day) median	0.0 (0.0-4.0)	0.0 (0.0-2.8)
OTD (day) median (25%-75%)	0.0 (0.0-5.0)	0.0 (0.0-5.0)
OTT (day) median (25%-75%)	1.0 (0.0-7.0)	1.5 (0.0-7.3)
ATT (days) median (25%-75%)	0.0 (0.0-3.0)	1.0 (0.0-2.0)
Treatment		
Direct surgery	12	27
Endovascular treatment	7	4
Observation	1	4
Rebleeding after admission		
Present	5	9
Absent	15	26
Death		
Present	2	5
Absent	18	29
Unknown (missing)	0	1

**Table 4:** MCA group GCS: Glasgow coma scale; OTA: onset-to-admission time; OTD; onset-to-diagnosis time; OTT: onset-to-treatment time; ATT: admission-to-treatment time; MCA: middle cerebral artery; N/A: not available.

Variable	MCA	
	Present (11)	Absent (44)
Age median (25%-75%)	46.0 (42.0-60.0)	51.0 (39.5-61.0)
Sex (male)	6	13
GCS at admission	15.0 (7.0-15.0)	14.0 (4.0-15.0)
Prehospital head trauma		
Present	1	1
Absent	8	30
Unknown (missing)	2	13
Episode indicating the rupture of aneurysm		
Present	9	41
Absent	2	2
Unknown (missing)	0	1
Prehospital rebleed		
Present	2	13
Absent	6	23
Unknown (missing)	3	8
Delayed diagnosis		
Present	2	10
Absent	9	34
Hematoma location		
Adjacent to the ruptured aneurysm	11	36
Far from the ruptured aneurysm	0	8
OTA (day) median	0.0 (0.0-8.5)	0.0 (0.0-1.0)
OTD (day) median (25%-75%)	1.5 (0.0-11.0)	0.0 (0.0-4.3)
OTT (day) median (25%-75%)	2.5 (0.0-9.0)	1.0 (0.0-7.0)
ATT (days) median (25%-75%)	0.0 (0.0-1.8)	0.0 (0.0-2.5)
Treatment		
Direct surgery	10	29
Endovascular treatment	0	11
Observation	1	4
Rebleeding after admission		
Present	3	11
Absent	8	33
Death		
Present	2	5
Absent	8	39
Unknown (missing)	1	0

**Table 5:** ACA group GCS: Glasgow coma scale; OTA: onset-to-admission time; OTD; onset-to-diagnosis time; OTT: onset-to-treatment time; ATT: admission-to-treatment time; ACA: anterior cerebral artery; N/A: not available.

Variable	ACA	
	Present (17)	Absent (38)
Age median (25%-75%)	54.0 (47.5-64)	46.5 (37.8-58.5)
Sex (male)	8	11
GCS at admission	14.0 (4.0-15.0)	15.0 (4.5-15.0)
Prehospital head trauma		
Present	1	1
Absent	11	27
Unknown (missing)	5	10
Episode indicating the rupture of aneurysm		
Present	16	34
Absent	0	4
Unknown (missing)	1	0
Prehospital rebleed		
Present	6	9
Absent	7	22
Unknown (missing)	4	17
Delayed diagnosis		
Present	5	7
Absent	12	31
Hematoma location		
Adjacent to the ruptured aneurysm	11	36
Far from the ruptured aneurysm	6	2
OTA (day) median	0.0 (0.0-0.0)	0.0 (0.0-6.0)
OTD (day) median (25%-75%)	0.0 (0.0-2.0)	0.0 (0.0-7.5)
OTT (day) median (25%-75%)	1.0 (0.0-6.0)	1.5 (0.0-9.3)
ATT (days) median (25%-75%)	1.0 (0.0-2.0)	0.0 (0.0-2.3)
Treatment		
Direct surgery	12	27
Endovascular treatment	2	9
Observation	3	2
Rebleeding after admission		
Present	5	9
Absent	12	29
Death		
Present	3	4
Absent	14	33
Unknown (missing)	0	1

**Table 6:** Pcom group GCS: Glasgow coma scale; OTA: onset-to-admission time; OTD; onset-to-diagnosis time; OTT: onset-to-treatment time; ATT: admission-to-treatment time; Pcom: posterior communicating artery; N/A: not available.

Variable	Pcom	
	Present (7)	Absent (48)
Age median (25%-75%)	39.0 (34.0-47.0)	51.0 (42.0-61.8)
Sex (male)	3	16
GCS at admission	15.0 (5.5-15.0)	14.0 (4.0-15.0)
Prehospital head trauma		
Present	0	2
Absent	6	32
Unknown (missing)	1	14
Episode indicating the rupture of aneurysm		
Present	7	43
Absent	0	4
Unknown (missing)	0	1
Prehospital rebleed		
Present	3	12
Absent	3	26
Unknown (missing)	1	10
Delayed diagnosis		
Present	2	10
Absent	5	38
Hematoma location		
Adjacent to the ruptured aneurysm	7	40
Far from the ruptured aneurysm	0	8
OTA (day) median	0.0 (0.0-7.0)	0.0 (0.0-1.0)
OTD (day) median (25%-75%)	1.0 (0.0-10.0)	0.0 (0.0-5.0)
OTT (day) median (25%-75%)	2.0 (1.0-10.0)	1.0 (0.0-6.3)
ATT (days) median (25%-75%)	1.0 (0.0-3.0)	0.0 (0.0-2.0)
Treatment		
Direct surgery	5	34
Endovascular treatment	2	9
Observation	0	5
Rebleeding after admission		
Present	1	13
Absent	6	35
Death		
Present	0	7
Absent	7	40
Unknown (missing)	0	1

From the results of a review of the literature, we discovered that low consciousness level on admission, delayed diagnosis of aneurysm rupture, distant hematoma relative to the ruptured aneurysm location, and rebleeding had high mortality rates in cases of ASDH. Furthermore, ACA aneurysms were more likely to present with a distant hematoma relative to the ruptured aneurysm location than other aneurysms, as in our case.

The rebleeding rate within 6 h of symptom onset was shown to be 3.66-fold higher than that more than 6 h after symptom onset [50], with the peak time of occurrence within 2 h of symptom onset [51]. The reported rebleeding rates within 24 h of symptom onset range between 4% and 13.6% [6,52]. In this study, 71.4% of patients who died experienced rebleeding. Furthermore, delayed diagnosis of aneurysm rupture and rebleeding was associated with increased mortality. A delayed diagnosis could lead to a high incidence of rebleeding, delayed diagnosis of pure ASDH would increase the mortality rates due to high rebleeding rates. Thus, preventing rebleeding might be the most important factor for reducing mortality in pure ASDH.

Following the analysis of aneurysm location characteristics, we discovered that the presence of ACA aneurysms had a more distant hematoma relative to the ruptured aneurysm location. Most ACA aneurysms (76.4%, 13/17) were distal aneurysms located in the interhemispheric fissure. As this area is restricted by the corpus callosum and bridging veins, it cannot be widened in cases of sudden acute bleeding, resulting in the spreading of the bleeding to the convexity space. In our case, after the intraoperative rupture, the bleeding spread from the interhemispheric to the convexity space (Figure 1e). Thus, patients with pure ASDHs without any episode of head trauma but with an episode of aneurysm rupture should be carefully evaluated to determine the bleeding origin. Furthermore, in assessing bleeding origin among patients with pure ASDH and distal ACA aneurysms, the possibility of distal ACA aneurysms as the bleeding site should be considered.

There were some limitations in this study. First, there was a risk of selection bias. Not all cases with pure ASDH were reported, and we included the reports in English and Japanese. Therefore, in this study, we did not perform statistical analysis. However, like pure ASDH, it was difficult to show the characteristics of rare diseases due to selection bias and a small sample size. A progressive statistical method that could reduce selection bias and the effect of sample size would be required in the review of rare cases in the future. Second, there was a risk of overestimation. We included the cases between 1981 and 2020. Spatial resonance of CT has improved over 40 years and all patients did not receive the test of cerebrospinal fluid on admission; therefore, slight SAH could be missed in the patients with a diagnosis of pure ASDH. Third, there were many missing data in this review. It is difficult to estimate how this issue affects the results of this study.

## Conclusions

This study showed a case of pure ASDH caused by a ruptured distal ACA aneurysm with the characteristics of a hematoma far from the ruptured site. A review of the literature indicates that delayed diagnosis of aneurysm rupture and rebleeding leads to poorer patient outcomes. In patients with ASDH without a clear history of head trauma, evaluation for intracranial artery aneurysms should be performed at the early stages of treatment, considering the possibility of a ruptured aneurysm. Furthermore, ruptured ACA aneurysms, particularly distal ACA aneurysms, may present with ASDH far from the bleeding site without SAH. Therefore, determining the cause of bleeding in these cases requires careful evaluation.
